# Cs[Tf_2_N]: a second polymorph with a layered structure

**DOI:** 10.1107/S2056989018004401

**Published:** 2018-03-23

**Authors:** Jared T. Stritzinger, Janelle E. Droessler, Brian L. Scott, George S. Goff

**Affiliations:** a Materials Physics and Applications Division, Associate Directorate for Experimental Physical Sciences, Los Alamos National Laboratory, Los Alamos, New Mexico, 87545, USA

**Keywords:** crystal structure, ionic liquid, bis­triflimide, layered structure

## Abstract

The structural determination of the ionic liquid, caesium bis­[(tri­fluoro­meth­yl)sulfon­yl]imide or poly[[μ_4_-bis­[(tri­fluoro­meth­yl)sulfon­yl]imido]caesium(I)], Cs[N(SO_2_CF_3_)_2_] or Cs[Tf_2_N], reveals a second polymorph that also crystallizes in a layer structure possessing monoclinic *P*2_1_/*c* symmetry at 120 K instead of *C*2/*c* for the known polymorph [Xue *et al.* (2002 ▸). *Solid State Sci.* **4**, 1535–1545]. The caesium ions in the cationic layers are coordinated by the sulfonyl groups of the bis­triflimide mol­ecules from anion layers while the tri­fluoro­methyl groups are oriented in the opposite direction, forming a non-polar surface separating the layers. The layer direction is (100).

## Abstract 2

The structure of the title ionic liquid is layered, with caesium and oxygen atoms forming the center of the layers and fluorine atoms forming the surface of the layers.

## Chemical context   

Recently, ionic liquids (IL) with melting points below 373 K, known as room temperature ionic liquids (RTIL), have emerged as a novel system that can be used to replace processes utilizing haza­rdous organic solvents and provide water-free environments (Welton, 1999 ▸). The exclusion of water from RTIL can be challenging as their ionic nature predisposes a hygroscopic nature, and even so-called hydro­phobic ILs can be difficult to dry (Francesco *et al.*, 2011 ▸). Reducing the solubility of water is possible by exchanging constituent ions of the IL for those that are less hydro­philic, but this often results in higher melting points or increased viscosity (Francesco *et al.*, 2011 ▸). The ability to change the physicochemical characteristics of ionic liquids has lead them to be praised as ‘tunable solvents’, but is often more of a challenging act of balancing physical properties.

The substitution of bis­(tri­fluoro­meth­yl)sulfon­yl)imide (bis­triflimide, Tf_2_N) as the anion in ILs offers a more hydro­phobic IL with lower melting point (Matsumoto *et al.*, 2002 ▸; Sun *et al.*, 1997 ▸). In general, anions of the triflate family are weakly coordinating when in the presence of other ligands, and inter­actions with metal ions may not be observed when in the presence of water. These weak inter­actions are due to the delocalization of charge across the mol­ecule. The structure of bis­triflimide also allows for multidentate coordination motifs when binding through the oxygen atoms, and often results in coordination of multiple metal cations. When Tf_2_N inter­actions expand beyond a single central metal atom, the ability to diffuse charge across a structure is highlighted (DesMarteau, 1995 ▸).

Cs[Tf_2_N] has a desirably low melting point of 398 K which is outside the conventional definition of a RTIL; however, this melting point is in the range of many well-known ILs while still being above the boiling point of water to enable con­venient drying (Hagiwara *et al.*, 2008 ▸; Scheuermeyer *et al.*, 2016 ▸). Previous reports of alkali metals and Tf_2_N include Cs[Tf_2_N], which presents as either an anhydrate or a variety of hydrates (Xue *et al.*, 2002 ▸). Some common structural similarities can be observed across the *A*[Tf_2_N]·*n*H_2_O (*A* = Li, Na, K, Rb, and Cs) series. The most notable feature is the formation of polar and non-polar regions that result from the coordination of multiple metal cations by the Tf_2_N^−^ ion. Each of the sulfonyl groups binds to a metal cation creating a polar chain that may extend to a layer, and orientates the tri­fluoro­methyl groups to create non-polar surfaces. Within the series, only the structures of Cs and K salts as anhydrates have been reported.

Our synthesis and analysis of Cs[Tf_2_N] has revealed a second layered polymorph set in *P*2_1_/c in addition to the previously reported structure in *C*2/c (Xue *et al.*, 2002 ▸).
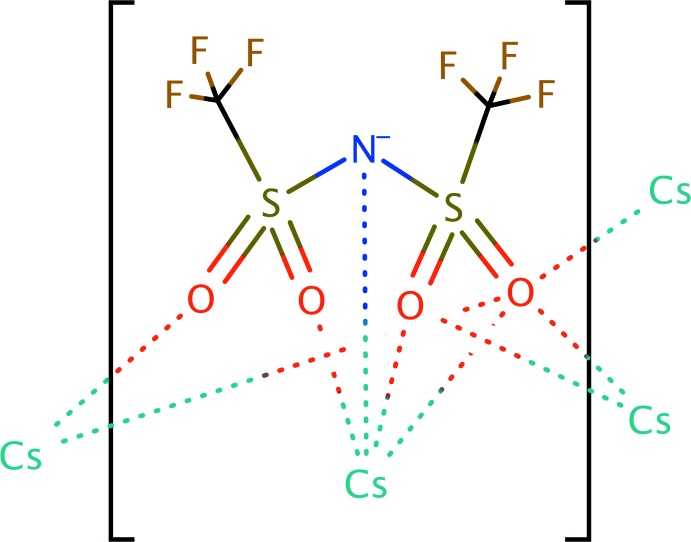


## Structural commentary   

**Figure 1:**
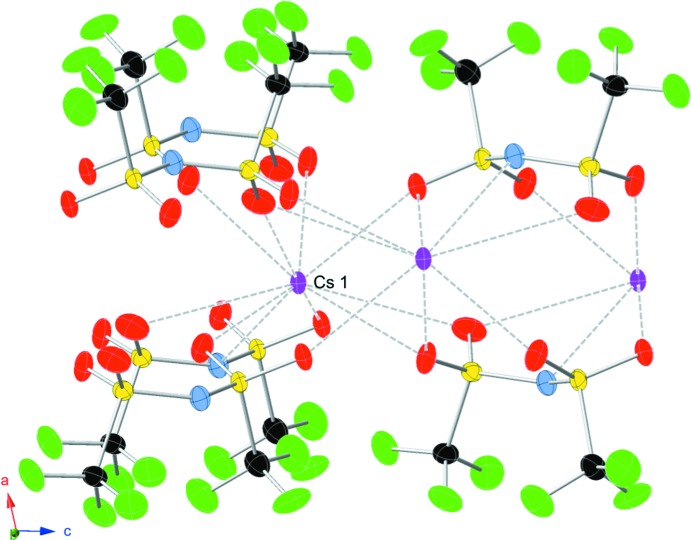
The coordination of the Cs^+^ cation by nine oxygen atoms and one nitro­gen atom of six different bis­triflimide anions coordinating above and three below, in a view slightly off the (100) plane. Other caesium cations are crystallographically equivalent to Cs1, and are shown to depict how the sheet extends. The displacement ellipsoids are drawn at the 50% probability function with the color scheme of caesium (purple), oxygen (red), sulfur (yellow), nitro­gen (blue), carbon (black), and fluorine (green).

The structure develops from the various ways in which six Tf_2_N mol­ecules coordinate the central 10-coordinate caesium cation (Fig. 1 ▸). The simplest coordination mode is monodentate, where one oxygen atom on one of the sulfonyl groups of the Tf_2_N mol­ecule coordinates to the caesium cation. The bidentate coordination mode has two motifs. In end-on coord­ination, both oxygen atoms of a single sulfonyl group coordinate with Cs^+^, while in side-on coordination one oxygen on each of the sulfonyl groups within a Tf_2_N mol­ecule coord­inate with Cs^+^. Two of the six distinct Tf_2_N mol­ecules exhibit the side-on coordination mode, and in one of them the nitro­gen atom of the Tf_2_N mol­ecule may come close enough to inter­act with the caesium cation. Examining the bond lengths in the coordination environment of the caesium cation, it is comprised of nine oxygen atoms ranging from 3.060 (2)–3.539 (3) Å and one inter­action with a nitro­gen atom of 3.280 (3) Å. These three different modes of Tf_2_N binding join the caesium cations together in a complex sheet with layers of tri­fluoro­methyl groups above and below (Fig. 1 ▸).

**Figure 2:**
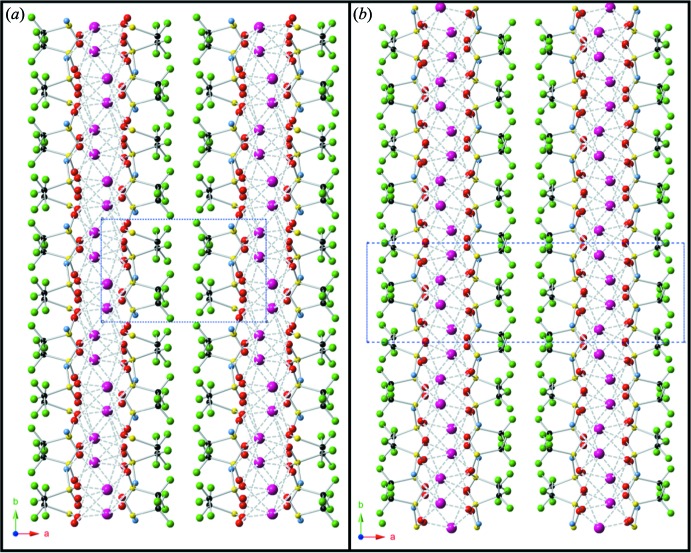
Ball and stick model view along [001] showing the layer of the structure arising from the hydro­phobic surfaces formed by orientation of the tri­fluoro­methyl groups, comparing (*a*) the unit cell of the stucture discussed in the paper set in *P*2_1_/*c* and (*b*) that of the previously reported structure set in *C*2/*c* (Xue *et al.*, 2002 ▸). Atoms are designated as caesium (purple), oxygen (red), sulfur (yellow), nitro­gen (blue), carbon (black), and fluorine (green).

As the Tf_2_N mol­ecule coordinates in the cis conformation, this orients the tri­fluoro­methyl groups in the opposite direction from the sulfonyl groups creating a layer of fluorine atoms. With this layer, tri­fluoro­methyl groups have an intra­molecular closest contact of 2.770 (4) Å and an inter­molecular closest contact of 2.970 (4) Å. There is a fluorine–fluorine closest contact length of 3.01 Å spanning the void between the non-polar surfaces of adjacent sheets in the layered structure. These layers are easily observed parallel to (100), Fig. 2 ▸. Examining the bis­triflimide mol­ecule, the S—N—S bond angle is 127.60 (17)° resulting in an intra­molecular carbon–carbon separation of 4.18 Å.

This structure of alternating layers of hydro­philic alkali metal cations bound by the sulfonyl groups and hydro­phobic layers of tri­fluoro­methyl groups closely matches the reported structures of K[Tf_2_N] and Cs[Tf_2_N] (Xue *et al.*, 2002 ▸). The noted deviations are in the coordination environment of the Cs^+^ cation. For the previously reported structure of Cs[Tf_2_N], the caesium coordination environment is also 10; however, the oxygen inter­actions are generally longer by about 0.05 Å, with Cs—O bonds ranging from 3.04 (1) to 3.65 (1) Å. The lone Cs—N bond is 3.39 (1) Å, which is considerably longer than the 3.280 (3) Å bond length observed in the current structure. This extension of bond lengths is reflected in the bis­triflimide mol­ecule where the S—N—S bond angle is contracted to 126.38 and the intra­molecular carbon–carbon separation is shortened to 4.08 Å. As the mol­ecule shifts, so does the orientation of the tri­fluoro­methyl groups, resulting in an intra­molecular closest contact of 2.72 Å and an inter­molecular closest contact of 2.96 Å. The shift also extends to the void between the non-polar surface of adjacent sheets, where fluorine–fluorine closest contacts are observed at 2.69 Å spanning the void. While the void space between layers appears reduced, the overall structure has a calculated density of 2.58 g cm^−3^ (Xue *et al.*, 2002 ▸), less dense than the calculated 2.65 g cm^−3^ of the more compact current structure.

**Figure 3:**
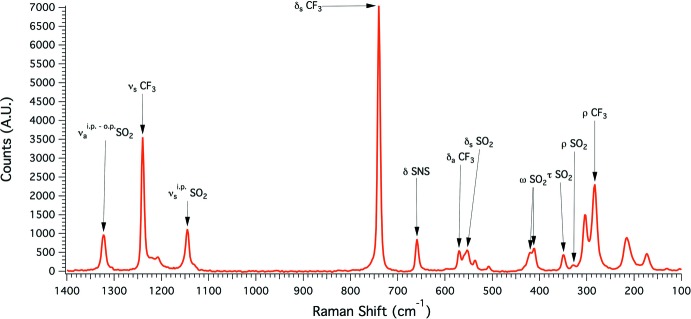
Raman spectra of Cs[Tf_2_N] with no other bands observed from 1400 cm^−1^ to 3200 cm^−1^. The additional bands at 535 and 507 cm^−1^ suggest the multiple SO_2_ bending modes associated with multiple coordination modes. Major band assignments are given with *ν* (stretching), *δ* (bending), *ω* (wagging), *τ* (twisting) and *ρ* (rocking) followed by the functional group. Planar designations are i.p.for in plane and o.p. for out of plane.

The Cs[Tf_2_N] purity was confirmed by melting point measurements that closely match literature values, showing an onset temperature of 397 K and complete melting at 399 K (Hagiwara *et al.*, 2008 ▸; Scheuermeyer *et al.*, 2016 ▸). Additional Raman analysis (Fig. 3 ▸), shows a number of features that closely match the reported spectra for Tf_2_N^−^ in water and solid-state measurements made on HTf_2_N, confirming the presence of the Tf_2_N mol­ecule (Rey *et al.*, 1998 ▸). To elucidate bands that signify inter­actions with metal cations, a comparison to the reported Raman spectra of La[Tf_2_N]_3_(H_2_O)_3_ (Bhatt *et al.*, 2005 ▸) was made. The major bands and assignments of all compounds in the comparisons are reported in Table 1 ▸. The additional bands observed at 535 and 507 cm^−1^ for Cs[Tf_2_N] suggest multiple SO_2_ bending modes associated with multiple coordination modes of Tf_2_N. In particular, the band at 507 cm^−1^ for Cs[Tf_2_N] matches with a 511 cm^−1^ band in La[Tf_2_N]_3_(H_2_O)_3_ suggesting a tentative assignment to a down-shift of the SO_2_ bending mode by the bidentate side-on coordination of the Tf_2_N mol­ecule, observed in both structures.

## Synthesis and crystallization   

All reagents were used as received without further purification. 20.281 g of caesium carbonate (Alfa Aesar, 99.9%) were dissolved in 20 ml of deionized water. 26.26 ml of 4.74 molar bis­triflimide acid (Alfa Aesar, 98.0%) were slowly added to the caesium carbonate solution, resulting in vigorous release of carbon dioxide. The solution was then placed in a sand bath at 403 K under stirring at approximately 100 rotations per minute. After four h, the temperature was reduced to 378 K. While the liquid cooled, the stirring deposited droplets of the ionic liquid on the sides of the beaker resulting in rapid crystallization. These crystals were harvested and suitable crystals were selected for diffraction. Yield was estimated at 95% based on mass.

### Experimental   

Raman measurements were collected using a Thermo Scientific DXRxi Raman Imaging Microscope. A 532 nm laser was focused on the sample surface through a 10x objective providing a spot size of 1 um and the collection consisted of 200 scans at 10 mW for 0.25 seconds each.

Melting point data were collected on a Büchi M-560, where two glass sample tubes were filled with 4–5mm of sample and the temperature was ramped at a rate of 0.5 K per minute.

## Refinement   

Crystal data, data collection and structure refinement details are summarized in Table 2 ▸.

## Supplementary Material

Crystal structure: contains datablock(s) I. DOI: 10.1107/S2056989018004401/wm5433sup1.cif

Structure factors: contains datablock(s) I. DOI: 10.1107/S2056989018004401/wm5433Isup2.hkl

CCDC reference: 1830211

Additional supporting information:  crystallographic information; 3D view; checkCIF report

## Figures and Tables

**Table 1:** Comparison of Raman modes in Tf_2_N compounds Comparison of observed Raman shifts in cm^−1^ from Tf_2_N-containing compounds. Band wavenumbers given in bold are unassigned and in italicized are from reanalysis of reported spectra for La[Tf_2_N]_3_(H_2_O)_3_. Major band assignments are given with ν (stretching), δ (bending), ω (wagging), τ (twisting) and ρ (rocking) and planar designations are i.p. for in plane and o.p. for out of plane.

Mode	Group	Tf_2_N^−^ in H_2_O	HTf_2_N	La[Tf_2_N]_3_(H_2_O)_3_	Cs[Tf_2_N]	
			**1456**			
			**1443**			
ν_a_ i.p.	SO_2_	1351				
ν_a_ o.p.	SO_2_	1332	1436	1316	1322	
			**1427**			
δ	NH		1332			
ν_s_	CF_3_	1239	1250	1243	1240	
ν_a_	CF_3_		1220			
		**1203**	**1208**	***1210***	**1206**	
ν_s_ i.p.	SO_2_	1131	1134	1148	1145	
δ_s_	CF_3_	744	765	754	740	
δ	SNS		634	*669*	659	
δ_a_ i.p.	SO_2_	594	583			
δ_a_	CF_3_	567	570	573	572	
			**555**			
δ_s_	SO_2_	551		554	553	
					535	
				*511*	507	
γ	NH		526			
			**495**	***444***		
ω	SO_2_	407, 401			420, 412	
			**380**			
τ	SO_2_	351		*356*	349	
		**339**				
ρ	SO_2_	325	335	332	328	
		**312**	**299**	**310**	**304**	
ν	MO			297?		
ρ	CF_3_	276		*288*	283	
			**264**	***241***		
			**210**		**215**	
			**202**		**173**	
			**185**		**130**	

**Table 2:** Experimental details

Crystal data	
Chemical formula	[Cs(C_2_F_6_NO_4_S_2_)]
*M*_r_	413.06
Crystal system, space group	Monoclinic, *P*2_1_/*c*
Temperature (K)	120
*a*, *b*, *c* (Å)	11.431 (4), 6.918 (2), 13.469 (4)
β (°)	103.686 (4)
*V* (Å^3^)	1035.0 (5)
*Z*	4
Radiation type	Mo *K*α
μ (mm^−1^)	4.07
Crystal size (mm)	0.40 × 0.35 × 0.33

Data collection	
Diffractometer	Bruker Photon 100
Absorption correction	Multi-scan (*SADABS*; Bruker, 2014 ▸)
*T*_min_, *T*_max_	0.507, 0.746
No. of measured, independent and observed [*I* > 2σ(*I*)] reflections	12467, 2354, 1980
*R*_int_	0.048
(sin θ/λ)_max_ (Å^−1^)	0.650

Refinement	
*R*[*F* ^2^ > 2σ(*F* ^2^)], *wR*(*F* ^2^), *S*	0.022, 0.058, 0.99
No. of reflections	2354
No. of parameters	145
Δρ_max_, Δρ_min_ (e Å^−3^)	0.84, −1.15

Computer programs: *APEX2* (Bruker, 2014 ▸), *SAINT-Plus* (Bruker, 2014 ▸), *SHELXT2014* (Sheldrick, 2015*a* ▸), *SHELXL2014* (Sheldrick, 2015*b* ▸), *CrystalMaker* (*CrystalMaker*, 2013 ▸).

## supplementary crystallographic information

### Crystal data

**Table:**

[Cs(C_2_F_6_NO_4_S_2_)]	*D*_x_ = 2.651 Mg m^−^^3^
*M**_r_* = 413.06	Melting point: 399 K
Monoclinic, *P*2_1_/*c*	Mo *K*α radiation, λ = 0.71073 Å
*a* = 11.431 (4) Å	Cell parameters from 12467 reflections
*b* = 6.918 (2) Å	θ = 3.1–27.5°
*c* = 13.469 (4) Å	µ = 4.07 mm^−^^1^
β = 103.686 (4)°	*T* = 120 K
*V* = 1035.0 (5) Å^3^	Block, colorless
*Z* = 4	0.40 × 0.35 × 0.33 mm
*F*(000) = 768	

### Data collection

**Table:**

Bruker Photon 100 diffractometer	2354 independent reflections
Radiation source: sealed tube	1980 reflections with *I* > 2σ(*I*)
Detector resolution: 0 pixels mm^-1^	*R*_int_ = 0.048
0.5 wide w/exposures scans	θ_max_ = 27.5°, θ_min_ = 3.1°
Absorption correction: multi-scan (*SADABS*; Bruker, 2014)	*h* = −14→14
*T*_min_ = 0.507, *T*_max_ = 0.746	*k* = −8→8
12467 measured reflections	*l* = −17→17

### Refinement

**Table:**

Refinement on *F*^2^	145 parameters
Least-squares matrix: full	0 restraints
*R*[*F*^2^ > 2σ(*F*^2^)] = 0.022	*w* = 1/[σ^2^(*F*_o_^2^) + (0.0256*P*)^2^ + 1.4815*P*] where *P* = (*F*_o_^2^ + 2*F*_c_^2^)/3
*wR*(*F*^2^) = 0.058	(Δ/σ)_max_ = 0.001
*S* = 0.99	Δρ_max_ = 0.84 e Å^−^^3^
2354 reflections	Δρ_min_ = −1.15 e Å^−^^3^

### Special details

**Table:**

Experimental. Single crystal data for [Cs][Tf_2_N] were collected on a Bruker D8 Quest diffractometer, with CMOS detector in shutterless mode. The crystal was cooled to 100 K employing an Oxford Cryostream liquid nitrogen cryostat. The diffractometer was equipped with graphite monochromatized MoKa (λ= 0.71073 Å) radiation. A hemisphere of data was collected using omega scans and 0.5° frame widths. Data collection and initial indexing and cell refinement were handled using APEX II1 (Bruker, 2014)software. Frame integration, including Lorentz-polarization corrections, and final cell parameter calculations were carried out using SAINT+ software (Bruker, 2014). The data were corrected for absorption using redundant reflections and the SADABS (Bruker, 2014)program. Decay of reflection intensity was not observed as monitored *via* analysis of redundant frames. The structure was solved using Direct methods and difference Fourier techniques.
Geometry. All esds (except the esd in the dihedral angle between two l.s. planes) are estimated using the full covariance matrix. The cell esds are taken into account individually in the estimation of esds in distances, angles and torsion angles; correlations between esds in cell parameters are only used when they are defined by crystal symmetry. An approximate (isotropic) treatment of cell esds is used for estimating esds involving l.s. planes.

### Fractional atomic coordinates and isotropic or equivalent isotropic displacement parameters (Å^2^)

**Table:**

	*x*	*y*	*z*	*U*_iso_*/*U*_eq_	
Cs1	−0.03866 (2)	0.63206 (3)	0.14267 (2)	0.01618 (7)	
S1	−0.19100 (7)	1.13281 (11)	0.03845 (6)	0.01796 (16)	
S2	−0.19461 (7)	0.61691 (11)	0.37165 (6)	0.01711 (16)	
F3	−0.3349 (2)	1.3866 (4)	0.0842 (2)	0.0490 (7)	
O1	−0.1407 (2)	1.0987 (4)	0.14485 (18)	0.0335 (6)	
F6	−0.4204 (2)	0.5355 (4)	0.29152 (18)	0.0473 (7)	
F2	−0.4023 (2)	1.0936 (4)	0.0776 (2)	0.0523 (7)	
F1	−0.4044 (2)	1.2485 (4)	−0.06094 (18)	0.0451 (6)	
F5	−0.3425 (2)	0.7475 (4)	0.21036 (16)	0.0419 (6)	
O4	−0.1614 (2)	0.4598 (4)	0.31460 (17)	0.0282 (6)	
F4	−0.3890 (2)	0.8228 (4)	0.3522 (2)	0.0512 (7)	
N1	−0.2092 (3)	0.5687 (4)	0.48229 (19)	0.0198 (6)	
O2	−0.1390 (2)	1.2826 (4)	−0.01035 (19)	0.0301 (6)	
O3	−0.1285 (2)	0.7926 (4)	0.3755 (2)	0.0355 (6)	
C2	−0.3470 (3)	0.6835 (6)	0.3026 (3)	0.0286 (8)	
C1	−0.3433 (3)	1.2186 (5)	0.0345 (3)	0.0284 (8)	

### Atomic displacement parameters (Å^2^)

**Table:**

	*U*^11^	*U*^22^	*U*^33^	*U*^12^	*U*^13^	*U*^23^
Cs1	0.02257 (12)	0.01463 (11)	0.01149 (10)	−0.00018 (7)	0.00432 (7)	0.00054 (7)
S1	0.0186 (4)	0.0238 (4)	0.0111 (3)	−0.0036 (3)	0.0026 (3)	−0.0028 (3)
S2	0.0171 (4)	0.0168 (4)	0.0171 (4)	−0.0003 (3)	0.0035 (3)	0.0026 (3)
F3	0.0498 (15)	0.0461 (15)	0.0501 (15)	0.0090 (12)	0.0098 (12)	−0.0265 (12)
O1	0.0318 (14)	0.0543 (17)	0.0119 (11)	0.0032 (13)	0.0004 (10)	−0.0044 (11)
F6	0.0293 (13)	0.0630 (17)	0.0413 (14)	−0.0197 (12)	−0.0084 (10)	0.0121 (12)
F2	0.0309 (13)	0.0638 (18)	0.0702 (19)	−0.0044 (12)	0.0278 (13)	0.0039 (14)
F1	0.0422 (14)	0.0450 (14)	0.0385 (13)	0.0177 (11)	−0.0094 (11)	−0.0069 (11)
F5	0.0439 (14)	0.0521 (15)	0.0249 (11)	0.0025 (12)	−0.0017 (10)	0.0189 (11)
O4	0.0422 (15)	0.0271 (13)	0.0193 (12)	0.0111 (11)	0.0157 (11)	0.0058 (10)
F4	0.0458 (15)	0.0580 (16)	0.0496 (15)	0.0322 (13)	0.0103 (12)	0.0048 (13)
N1	0.0249 (15)	0.0184 (13)	0.0154 (13)	0.0017 (11)	0.0034 (11)	−0.0029 (10)
O2	0.0414 (15)	0.0269 (13)	0.0266 (13)	−0.0148 (11)	0.0170 (11)	−0.0097 (10)
O3	0.0315 (15)	0.0251 (13)	0.0462 (16)	−0.0104 (11)	0.0019 (12)	0.0091 (12)
C2	0.0229 (18)	0.0355 (19)	0.0259 (18)	0.0031 (15)	0.0029 (14)	0.0092 (15)
C1	0.0284 (19)	0.0291 (19)	0.0257 (18)	0.0036 (15)	0.0022 (15)	−0.0061 (14)

### Geometric parameters (Å, º)

**Table:**

Cs1—O2^i^	3.060 (2)	S2—N1	1.574 (3)
Cs1—O3^ii^	3.074 (3)	S2—C2	1.829 (4)
Cs1—O1^ii^	3.110 (2)	S2—Cs1^vii^	4.0548 (12)
Cs1—O4^iii^	3.174 (3)	F3—C1	1.333 (4)
Cs1—O2^iv^	3.208 (2)	F3—Cs1^vi^	3.703 (3)
Cs1—O4	3.207 (2)	O1—Cs1^iii^	3.110 (2)
Cs1—N1^v^	3.280 (3)	F6—C2	1.310 (5)
Cs1—O1	3.435 (3)	F2—C1	1.314 (5)
Cs1—O3^v^	3.539 (3)	F1—C1	1.326 (4)
Cs1—O3	3.694 (3)	F5—C2	1.331 (4)
Cs1—F3^iv^	3.703 (3)	O4—Cs1^ii^	3.175 (3)
Cs1—S2	3.9118 (12)	F4—C2	1.326 (5)
S1—O1	1.432 (2)	N1—S1^vii^	1.576 (3)
S1—O2	1.430 (3)	N1—Cs1^vii^	3.280 (3)
S1—N1^v^	1.576 (3)	O2—Cs1^i^	3.060 (2)
S1—C1	1.828 (4)	O2—Cs1^vi^	3.208 (2)
S1—Cs1^vi^	3.9719 (12)	O3—Cs1^iii^	3.074 (3)
S2—O3	1.426 (3)	O3—Cs1^vii^	3.539 (3)
S2—O4	1.433 (2)		
			
O2^i^—Cs1—O3^ii^	65.81 (8)	O3—Cs1—S2	21.36 (4)
O2^i^—Cs1—O1^ii^	99.58 (7)	F3^iv^—Cs1—S2	65.85 (4)
O3^ii^—Cs1—O1^ii^	74.33 (7)	O1—S1—O2	117.87 (17)
O2^i^—Cs1—O4^iii^	54.48 (6)	O1—S1—N1^v^	108.10 (16)
O3^ii^—Cs1—O4^iii^	97.22 (8)	O2—S1—N1^v^	116.27 (14)
O1^ii^—Cs1—O4^iii^	66.20 (7)	O1—S1—C1	103.70 (16)
O2^i^—Cs1—O2^iv^	87.52 (7)	O2—S1—C1	104.35 (17)
O3^ii^—Cs1—O2^iv^	60.48 (7)	N1^v^—S1—C1	104.86 (16)
O1^ii^—Cs1—O2^iv^	126.37 (7)	O1—S1—Cs1^vi^	75.58 (12)
O4^iii^—Cs1—O2^iv^	141.95 (6)	O2—S1—Cs1^vi^	48.47 (10)
O2^i^—Cs1—O4	162.97 (7)	N1^v^—S1—Cs1^vi^	159.36 (11)
O3^ii^—Cs1—O4	99.20 (7)	C1—S1—Cs1^vi^	93.60 (12)
O1^ii^—Cs1—O4	67.27 (7)	O1—S1—Cs1	57.66 (12)
O4^iii^—Cs1—O4	123.62 (5)	O2—S1—Cs1	126.74 (12)
O2^iv^—Cs1—O4	91.89 (7)	N1^v^—S1—Cs1	52.89 (10)
O2^i^—Cs1—N1^v^	82.39 (7)	C1—S1—Cs1	128.83 (13)
O3^ii^—Cs1—N1^v^	135.52 (7)	Cs1^vi^—S1—Cs1	120.90 (3)
O1^ii^—Cs1—N1^v^	144.35 (7)	O3—S2—O4	117.41 (17)
O4^iii^—Cs1—N1^v^	87.65 (7)	O3—S2—N1	108.66 (16)
O2^iv^—Cs1—N1^v^	89.20 (7)	O4—S2—N1	116.61 (14)
O4—Cs1—N1^v^	114.62 (7)	O3—S2—C2	103.71 (17)
O2^i^—Cs1—O1	95.58 (7)	O4—S2—C2	105.06 (17)
O3^ii^—Cs1—O1	159.64 (7)	N1—S2—C2	103.49 (16)
O1^ii^—Cs1—O1	102.31 (7)	O3—S2—Cs1	70.70 (12)
O4^iii^—Cs1—O1	63.81 (7)	O4—S2—Cs1	50.92 (10)
O2^iv^—Cs1—O1	130.03 (7)	N1—S2—Cs1	157.27 (11)
O4—Cs1—O1	97.80 (6)	C2—S2—Cs1	98.57 (12)
N1^v^—Cs1—O1	42.47 (6)	O3—S2—Cs1^vii^	59.03 (12)
O2^i^—Cs1—O3^v^	56.78 (7)	O4—S2—Cs1^vii^	133.75 (11)
O3^ii^—Cs1—O3^v^	93.89 (6)	N1—S2—Cs1^vii^	50.27 (10)
O1^ii^—Cs1—O3^v^	156.36 (7)	C2—S2—Cs1^vii^	120.87 (13)
O4^iii^—Cs1—O3^v^	95.87 (6)	Cs1—S2—Cs1^vii^	120.87 (3)
O2^iv^—Cs1—O3^v^	58.95 (7)	C1—F3—Cs1^vi^	117.4 (2)
O4—Cs1—O3^v^	135.86 (7)	S1—O1—Cs1^iii^	158.44 (17)
N1^v^—Cs1—O3^v^	41.69 (6)	S1—O1—Cs1	101.72 (13)
O1—Cs1—O3^v^	81.53 (6)	Cs1^iii^—O1—Cs1	85.77 (6)
O2^i^—Cs1—O3	142.43 (6)	S2—O4—Cs1^ii^	135.68 (14)
O3^ii^—Cs1—O3	126.88 (6)	S2—O4—Cs1	108.79 (12)
O1^ii^—Cs1—O3	59.74 (7)	Cs1^ii^—O4—Cs1	88.69 (6)
O4^iii^—Cs1—O3	87.95 (6)	S2—N1—S1^vii^	127.60 (17)
O2^iv^—Cs1—O3	130.02 (6)	S2—N1—Cs1^vii^	108.07 (13)
O4—Cs1—O3	40.70 (6)	S1^vii^—N1—Cs1^vii^	104.58 (12)
N1^v^—Cs1—O3	97.36 (7)	S1—O2—Cs1^i^	144.39 (15)
O1—Cs1—O3	63.07 (6)	S1—O2—Cs1^vi^	112.04 (12)
O3^v^—Cs1—O3	138.32 (6)	Cs1^i^—O2—Cs1^vi^	92.48 (7)
O2^i^—Cs1—F3^iv^	131.63 (6)	S2—O3—Cs1^iii^	169.31 (18)
O3^ii^—Cs1—F3^iv^	100.78 (7)	S2—O3—Cs1^vii^	100.76 (13)
O1^ii^—Cs1—F3^iv^	122.16 (7)	Cs1^iii^—O3—Cs1^vii^	86.11 (6)
O4^iii^—Cs1—F3^iv^	161.69 (6)	S2—O3—Cs1	87.94 (13)
O2^iv^—Cs1—F3^iv^	49.29 (6)	Cs1^iii^—O3—Cs1	81.90 (6)
O4—Cs1—F3^iv^	56.56 (7)	Cs1^vii^—O3—Cs1	146.66 (8)
N1^v^—Cs1—F3^iv^	77.18 (7)	F6—C2—F5	108.6 (3)
O1—Cs1—F3^iv^	97.90 (6)	F6—C2—F4	109.1 (3)
O3^v^—Cs1—F3^iv^	79.67 (6)	F5—C2—F4	109.1 (3)
O3—Cs1—F3^iv^	83.94 (6)	F6—C2—S2	111.6 (3)
O2^i^—Cs1—S2	162.46 (5)	F5—C2—S2	108.4 (2)
O3^ii^—Cs1—S2	116.76 (6)	F4—C2—S2	110.0 (3)
O1^ii^—Cs1—S2	66.21 (6)	F2—C1—F1	108.9 (3)
O4^iii^—Cs1—S2	108.62 (4)	F2—C1—F3	109.5 (3)
O2^iv^—Cs1—S2	109.03 (5)	F1—C1—F3	108.0 (3)
O4—Cs1—S2	20.30 (5)	F2—C1—S1	111.0 (3)
N1^v^—Cs1—S2	103.02 (5)	F1—C1—S1	111.0 (3)
O1—Cs1—S2	78.43 (4)	F3—C1—S1	108.3 (3)
O3^v^—Cs1—S2	136.79 (5)		

Symmetry codes: (i) −*x*, −*y*+2, −*z*; (ii) −*x*, *y*−1/2, −*z*+1/2; (iii) −*x*, *y*+1/2, −*z*+1/2; (iv) *x*, *y*−1, *z*; (v) *x*, −*y*+3/2, *z*−1/2; (vi) *x*, *y*+1, *z*; (vii) *x*, −*y*+3/2, *z*+1/2.
